# Radial left ventricular dyssynchrony by speckle tracking in apical versus non apical right ventricular pacing- evidence of dyssynchrony on medium term follow up

**DOI:** 10.15171/jcvtr.2016.04

**Published:** 2016-03-14

**Authors:** Dinesh Choudhary, Amit Kumar Chaurasia, S Mahesh Kumar, Ajeet Arulkumar, Anees Thajudeen, Narayanan Namboodiri, G Sanjay, SP Abhilash, VK Ajitkumar, Tharakan JA

**Affiliations:** Department of Cardiology, Sree Chitra Tirunal Institute for Medical Sciences and Technology, Trivandrum 695 011, Kerala, India

**Keywords:** Left Ventricle, Artificial Cardiac Pacing, Ventricular Function

## Abstract

***Introduction:*** To study effects of various sites of right ventricular pacing lead implantation on left ventricular function by 2-dimensional (2D) speckle tracking for radial strain and LV dyssynchrony.

***Methods:*** This was retrospective prospective study. Fifteen patients each with right ventricular (RV) apical (RV apex and apical septum) and non-apical (mid septal and low right ventricular outflow tract [RVOT]) were programmed to obtain 100% ventricular pacing for evaluation by echo. Location and orientation of lead tip was noted and archived by fluoroscopy. Electrocardiography (ECG) was archived and 2D echo radial dyssynchrony was calculated.

***Results:*** The baseline data was similar between two groups. Intraventricular dyssynchrony was significantly more in apical location as compared to non-apical location (radial dyssynchrony: 108.2 ± 50.2 vs. 50.5 ± 24, P < 0.001; septal to posterior wall delay [SLWD] 63.5 ± 27.5 vs. 34 ± 10.7, *P* < 0.001, SPWD 112.5 ± 58.1 vs. 62.7 ± 12.1, *P* = 0.003). The left ventricular ejection fraction was decreased more in apical location than non apical location. Interventricular dyssynchrony was more in apical group but was not statistically significant. The QRS duration, QTc and lead thresholds were higher in apical group but not statistically significant.

***Conclusion:*** Pacing in non apical location (RV mid septum or low RVOT) is associated with less dyssynchrony by specific measures like 2D radial strain and correlates with better ventricular function in long term.

## Introduction

Cardiac pacing is treatment of choice in the management of patients with bradyarrhythmias and drug refractory heart failure in the form of cardiac resynchronization therapy (CRT).^1,2^ Traditional site of pacing is right ventricular (RV) apex, however; adverse left ventricular remodelling and dyssynchrony could be the limiting factor. The relationship between RV apical pacing and mechanical dyssynchrony has been studied in various trials.^3-11^ Though they provide good patho-physiologic insight, they are not sufficient to recommend an optimal RV pacing site mainly because of the variability in lead insertion site. We planned this study to answer some of these questions by identifying the lead position on fluoroscopy and then comparing electrocardiography (ECG) and dyssynchrony parameters with ventricular function on medium term follow up.

2-dimensional (2D) strain based speckle tracking which evaluates circumferential strain (CS), longitudinal strain (LS), and radial strain (RS) by self tracking of myocardial segments allows better advantage in comparison to tissue Doppler imaging as it does not depend on Doppler angle and also monitors strains in two dimension rather than one dimension, thus increasing reproducibility and accuracy. Therefore, we used this technique in our study.

## Materials and Methods

This was a retrospective-prospective study, in which we retrospectively selected 30 patients with single chamber pacemaker (VVI) with RV apical (RV apex and apical septum) and RV non apical (mid septal and low right ventricular outflow tract [RVOT]) pacing and who satisfied the inclusion criteria and prospectively followed them for 40 months and evaluated them for ventricular function and dyssynchrony parameters.

### Inclusion and exclusion criteria

Patients with normal pacemaker function, without any structural heart disease, who were more than 18 years, had ≥ 80% pacing at follow up (by pacemaker interrogation or in 2 ECGs taken at two different times) and who provided consent for the study were included in the study. Patients who refused consent, had coronary or structural heart disease, cardiomyopathies or any form of cardiac surgery were excluded from the study.

### Procedures and data collection

**Figure 1:**
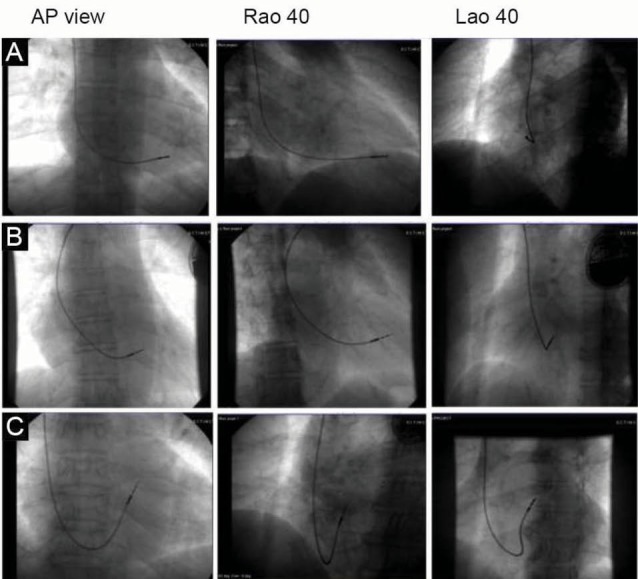
Fluoroscopic images of various lead locations of RV pacing (A) apical position (B) mid septal position (C) RVOT position.

The date of pacemaker implantation, indication for implantation, lead implant location, lead parameters and hardware used while implanting the pacemaker were noted from patients’ records. Fluoroscopy was performed after obtaining patients consent. The lead location and orientation of the lead tip were noted and archived by fluoroscopy (AP, RAO 40, LAO 40 and left lateral views) (Figure 1). A 12 lead ECG was performed and archived. Longest QRS, QTc (by using Bazett’s formula (QT/√RR interval) duration and QRS axis were calculated for all patients. Interrogation of pacemaker was done to see pacemaker function, percentage of pacing and to check various lead parameters (threshold and lead impedance). Following which, 100% ventricular pacing was programmed for evaluation by echocardiography.

### Evaluation of radial strain and dyssynchrony

**Figure 2:**
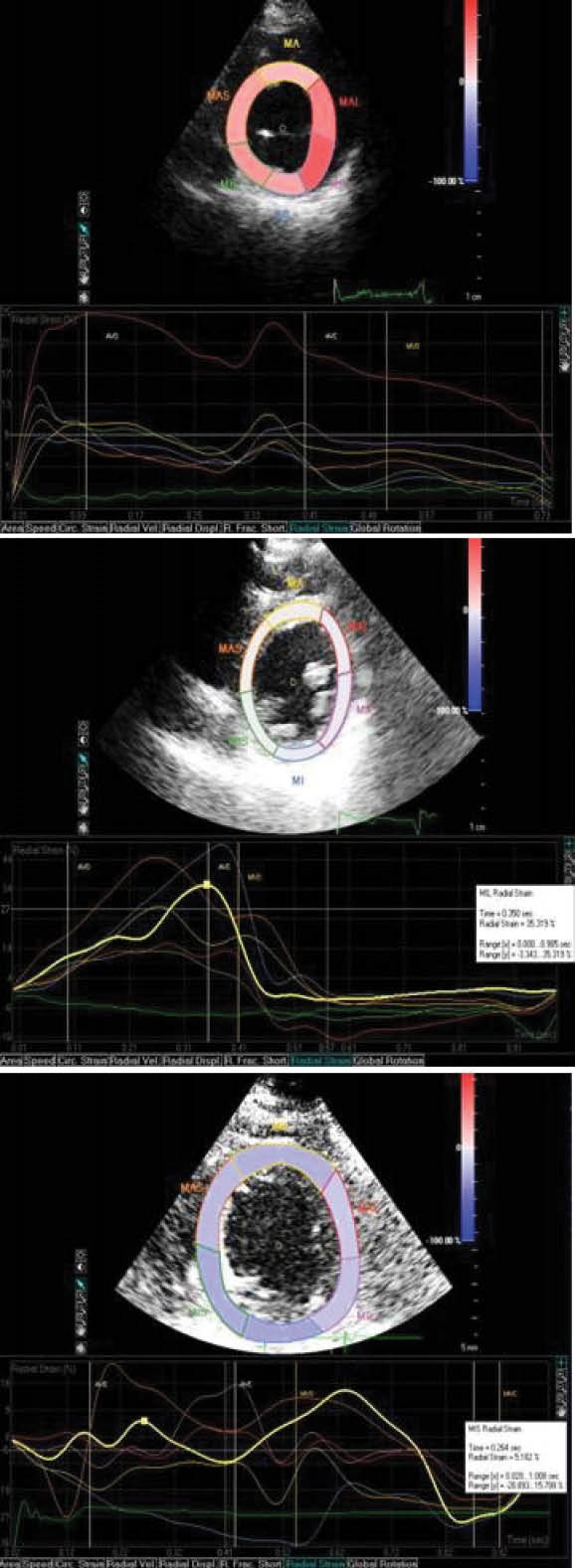
Echocardiographic images of radial dyssynchrony showing (A) No significant radial dyssynchrony on 2D speckle strain analysis; (B) Mild radial dyssynchrony on 2d speckle strain analysis; (c) significant radial dyssynchrony on 2D speckle strain analysis.

When 100% ventricular pacing was confirmed, echocardiography was performed with PHILIPS iE 33 (Philips Healthcare, Andover, USA). First, the position of the lead was confirmed and then 2D short axis images of the left ventricle at the mid papillary muscle level with breath held in expiration was obtained, following which four loops with frame rate of 50 to 80 per second was recorded. After this all the analysis was done offline using Q-lab software for PHILIPS iE 33 machine. During offline evaluation, first, the endocar­dium was traced manually at the end-systolic frame and divided into 6 seg­ments and then strain curves for each segment was constructed. After this, the time to peak radial strain of each segment was measured. And then finally, the absolute time interval of peak strain between anteroseptum and posterior segment was calculated along with radial dyssynchrony (Figure 2). We also measured intraventricular dyssynchrony (septal to posterior wall delay [SPWD] by M-mode, septal to lateral wall delay [SLWD] by tissue Doppler imaging [TDI]) and interventricular dyssynchrony (difference in the electromechanical delay [Q-aortic ejection and Q-pulmonary artery ejection]).

### Statistical analysis

All statistical analysis was done using SPSS 14 software (SPSS Inc, Chicago, Illinois). Categorical data were analyzed using chi-square test. Continuous data were analyzed by student *t* test and presented as mean ± SD. *P*<0.05 was considered as significant. Pearson correlation coefficient test was used to see correlation between various dyssynchrony indices.

## Results

Thirty patients; 15 with RV apical (RV apex and apical septum) and 15 with RV non apical (mid septum and low RVOT) pacing having single chamber pacemaker with VVI pacing were studied. 7 patients (46.6%) were males and 8 (53.3%) were females (Table 1) in each group. The mean age of males in apical group was 63 ± 21.9 years and in non apical group was 60.7 ± 16.5 years (*P=*0.829); while the mean age of females was 61.1 ± 7.3 years vs. 63 ± 18.7 years respectively (*P=*0.796). Overall the mean age of apical and non apical pacing was 62.0 ±15.3 years vs. 61.9 ±17.1 years respectively (*P=*0.991).

**Table 1:** Baseline characteristics of patients

	**Apical (n=15)**	**Non-apical (n=15)**
Male (n)	46.7% (7)	46.7% (7)
Age (y) (mean ± SD)	62.0 ± 15.3	61.9 ±17.1
Site of PP lead		
Apex (n)	33.3% (5)	NA
Apical Septum (n)	66.7% (10)	NA
Mid Septum (n)	NA	46.7% (7)
RVOT (n)	NA	53.3% (8)
Diagnosis		
CHB (n)	53.3% (8)	86.07% (13)
High grade AV block (n)	13.3% (2)	6.7% (1)
SSS (n)	33.3% (5)	6.7% (1)
NYHA FC I (n)	86.7% (13)	93.3% (14)
NYHA FC II (n)	13.3% (2)	6.7% (1)
DM (n)	26.7% (4)	46.7% (7)
HTN (n)	60% (9)	73.3% (11)
DLP (n)	33.3% (5)	60% (9)
Lead parameters		
Basal threshold	0.6 ± 0.2	0.6 ± 0.1
F/U threshold	0.9 ± 0.4	0.8 ± 0.2
Basal impedance	847.7 ± 195.4	774.7 ± 112.7
F/U impedance	531.1 ± 147.7	537.5 ± 105.3
% Pacing	92.9 ± 8.4	94.6 ± 7.9
% Change in threshold	51.8 ± 57.0	50.9 ± 48.2
% Change in Impedance	34.8 ± 21.1	29.1 ± 16.9
ECG parameters		
ORS axis	- 69.46 ± 14.86	+ 76.76 ± 40.59
ORS duration (ms)	165.7 ± 18.7	158.7 ± 15.4
QTc (ms)	466.1 ± 22.9	463.5 ± 23.0

Abbreviations: PP, Permanent pacemaker; A, apex; AS, apical septum; RVOT, right ventricular outflow tract; CHB, complete heart block ; AV, Atrioventricular; FC, functional class; F/U, Follow-up; DM, diabetes mellitus; HTN, hypertension; DLP, dyslipidemia; CAD, Coronary artery disease.

Five patients in apical group had pacing lead in RV apex position and 10 patients had in apical septal position; whereas in non apical group 7 patients had pacing lead in mid septum and 8 patients at low RVOT pacing (Table 1). At baseline CHB was present in 8 patients in apical group and in 13 in non apical group, high grade AV block in 2 and 1 respectively and sick sinus syndrome (SSS) in 5 and 1 patients respectively. At follow up 2 patients in apical group and 1 patient in non apical group were in NYHA class 2. Rest of the patients in both groups were in NYHA class 1. There was no statistically significant difference in other baseline characteristics (risk factors of coronary artery disease, smoking, diabetes mellitus (DM), hypertension, dyslipidemia, family history of CAD) (Table 1).

Mean follow up in apical and non apical groups was 38.2 ± 20 months and 32.6 ± 22.1 months respectively. During this period 92.9 ± 8.4 % of the time patients in apical pacing group had ventricular pacing whereas in non apical pacing it was 94.6 ± 7.9 % respectively (*P=*0.575). There was no significant difference in lead impedance, lead threshold, and percentage change in lead impedance in both the groups at baseline and on follow up (Table 1).

The mean QRS duration was 165.7 ± 18.7 ms in apical pacing group and 158.7 ± 15.4 ms in non apical pacing group (*P=*0.273). QTc duration was 466.1 ± 22.9 ms and 463.5 ± 23 ms respectively (*P=*0.759). Mean QRS Axis was -69.46 degrees in apical group and +76.76 degrees in non apical group (Table 1).

The left ventricular ejection fraction was decreased more in apical location (mean drop of 6.7% from baseline; *P=*0.06*)* than non apical location (mean drop of 1.3% from baseline, *P=0*.68). But there was no significant difference among percent change in EF when both groups compared to each other (*P=*0.278; Table 2).

**Table 2:** Echocardiography variable in apical and non-apical groups

	**Apical**			**Non-apical**		***P***
	**Pre**	**Post**	***P***	**Pre**	**Post**	
LVIDD (mm)	51.1 ± 7	48.3 ± 6	0.134	48.7 ± 7.8	47.2 ± 5.6	0.209
LVIDS (mm)	32.1 ± 5.4	30.4 ± 6.4	0.415	31.0 ± 6.0	28.8 ± 8.9	0.091
EF (%)	73.1 ± 8.8	66.4 ± 10.0	0.064	70.1 ± 8.9	68.9 ± 10.0	0.686
LA (mm)	35.3 ± 3.6	35.7 ± 3.9	0.771	33.7 ± 5.3	34.7 ± 4.3	0.438

Abbreviations: LVIDD, Left ventricular internal dimension diastole; LVIDS, Left ventricular internal dimension systole; EF, Ejection Fraction; LA, Left atrium.

Intraventricular dyssynchrony was significantly more in the apical location as compared to non-apical location (radial dyssynchrony was measured as time difference between peak strain of the anteroseptum and posterior/inferior wall: 108.2 ± 50.2 vs. 50.5 ± 24 ms, *P=*0.001; SLWD 63.5 ±27.5 vs. 34 ±10.7 ms, *P=*0.001, SPWD 112.5 ± 58.1 vs. 62.7 ± 12.1 ms, *P=*0.003). There was no significant difference between mean peak strain in the 2 groups (25.6 ± 9.6 vs. 22.7 ± 10.2; *P=*0.426) though it was less in non apical group. Interventricular dyssynchrony was also more in apical group but not statistically significant (Qao-Qpo 43.4 ± 21.4 v/s 36.6 ± 36.6 ± 13.8; *P=*0.30) (Table 3).

**Table 3:** Comparison of dyssynchrony variables in different groups

	**Apical**	**Non-Apical**	**P**
Radial dyssynchrony	108.2 ± 50.2	50.5 ± 24.0	0.0001
Peak strain	26.6 ± 9.6	22.7 ± 10.2	0.426
SPW delay	112.5 ± 58.1	62.7 ± 12.1	0.003
SL delay	63.5 ± 27.5	34.0 ± 10.7	0.001
QaO	144.8 ± 26.9	141.8 ± 22.8	0.744
QaO-Qpo	43.4 ± 21.4	36.6 ± 13.8	0.309
RR	968.2 ± 91.5	978.0 ± 85.2	0.764

Abbreviations: SPW; Septal to posterior wall; SLW; Septal to lateral wall; Qao; Q aortic ejection; Qpo; Q pulmonary ejection; RR; RR interval.

Pearson correlation showed that radial dyssynchrony was positively correlated with SPW delay in apical (r: 0.546; *P=*0.035) and non apical group (r: 0.121; *P=*0.668) though it was significant only in apical group. It was positively correlated with SL delay in apical (r: 0.477; *P=*0.072) and negatively in non-apical group (r: -0.011; *P=*0.970) but it was not statistically significant (Table 4).

**Table 4:** Pearson correlation between radial dyssynchrony and SPW delay, SL delay

	**Apical**		**Non-apical**	
	**r**	***P***	**r**	***P***
SPW delay	0.546	0.035	0.121	0.668
SL delay	0.477	0.072	-0.011	0.970

## Discussion

The site of RV pacing has been a subject of controversy. A number of studies have looked into the association of RV apical pacing and mechanical dyssynchrony and its deleterious effect on ventricular function.^3-11^ However, these studies have been confounded by the fact that the site of lead has been variable. In this study, we tried to be precise by taking as much fluoroscopy view as possible so that the position could be well defined. We found a significant beneficial effect of RV outflow-tract pacing in comparison to apical . Although the result shows significant benefit; due to small sample size this study may be regarded as a pilot study and further more work be done in this direction.

There was no significant difference between 2 groups regarding baseline mean age, DM, hypertension, dyslipidemia, smoking. The duration of study has been a concern in pacemaker studies as short duration may not be able to mask the true deleterious effect. Tse et al^7^ were not able to show any deleterious effect between RVOT pacing and RV apex pacing at 18 months; however, with increasing duration (7 years) Lewicka-Nowak et al^12^ were able to show significant drop in ejection fraction. Similarly, the result of other short term studies^13-15^ could also be confounded by the same problem. In our study, the mean duration of follow up was 38.2 months in apical group and 32.6 months in non-apical group which may be a considerable long time to unmask the deleterious effect.

As with Burri et al^16^ study we too did not find any difference in the lead threshold, lead resistance and percentage change of both parameters in both groups, both in the beginning as well as on follow up. We in our study found the mean QRS, QTc duration and left ventricular ejection fraction was not statistically significant in RV apical and non apical group. However, a meta-analysis done by de Cock et al^13^ and another trial by Victor et al^15^ showed shorter or near normal QRS duration was associated with better LV contraction and less LV dysfunction in RVOT and septal pacing than in apical pacing. Though difficult to explain it may be due to shorter duration of our study and small sample size. The intraventricular dyssynchrony was significantly more in apical pacing group than in non apical pacing group; however, the peak strain, and also the interventricular dyssynchrony was not significant in the two group. Pearson correlation showed significant positive correlation between radial dyssynchrony and SPW delay in apical pacing group. Though non significant, SL delay was positively correlated with radial dyssynchrony in apical pacing and negatively correlated in non apical pacing group. Favorable haemodynamics and less dyssynchrony was seen in RVOT and septal location pacing in comparison to apical location pacing in most trials^15-17^,and a meta analysis^13^; however, some contradicts too.^14,18^ More physiologic activation pattern of LV activation may have lead to less LV dyssynchrony in these trials. One study from Korea^19^ showed despite increase in QRS duration after pacing; M-mode, Doppler and TDI failed to show any difference in dyssynchrony parameters. 2D speckle tracking method for the dyssynchrony was not used in this study. A study from Japan^20^ that used speckle tracking method was able to show right ventricular septal (RVS) pacing preserves global left ventricular longitudinal function in comparison with RV Apical (RVA) pacing. They noted that due to heterogeneous RV apical pacing there was deterioration on LV longitudinal contraction and therefore, RV septal pacing could be a better pacing alternative when LV dyssynchrony and longitudinal LV function was concerned. Similarly, in our study too we found less radial dyssynchrony in non-apical location which may result in favorable hemodynamics and preserved LV function on long term follow up. However due to small sample size the other correlation may not have been significant. As noted by others^21^ tissue Doppler imaging and derived strain and strain rate measurements lacks reproducibility which may limit their use in clinical studies. The lack of reproducibility may be because of the dependency on Doppler angle for their measurement. Other factors like regional myocardial velocities that causes tethering effects from other myocardial segments and translational motion of the entire heart may play role. Due to avoidance of these limiting factor 2D speckle tracking technique for strain measurement may provide better information.^22-25^

Though recently published "*Right ventricular apical and high septal pacing to preserve left ventricular function (Protect Pace)*"^26^ study showed no deterioration in LV functions when RV apical pacing was compared to septal pacing at two years. However, in a subgroup analysis^27^ of the same patient inefficient dyssynchronous contraction and decrease in apical strain was seen with RV apical pacing which may lead to global LV function deterioration. There are two other studies that are underway (optimize RV selective site pacing clinical trial (Optimize RV) and Right Ventricular Apical versus Septal Pacing [RASP]) that may throw light on this problem.^28^

## Study limitation

The sample size is small and also the duration may not have been enough to study some factors that may have affected the study. However, we plan to take this study as a pilot study and extend this study by recruiting more patients for a longer duration so that a robust conclusion could be reached.

## Conclusion

Pacing in the non apical location (RV mid septum or low RVOT) is associated with less dyssynchrony by specific measures like 2D radial strain and may correlate with better ventricular function in the long term. However, longer duration follow up and larger sample size is required to confirm this finding.

## Ethical issues

The Study was approved by Institutional ethical committee.

## Competing interests

Authors declare no conflict of interest in this study.
